# Supplementary figures and images for: Maize (Zea mays L.) Nucleoskeletal Proteins Regulate Nuclear Envelope Remodeling and Function in Stomatal Complex Development and Pollen Viability
Source: Front Plant Sci. 2021 Feb 17;12:645218. doi: 10.3389/fpls.2021.645218 (PMC7925898; doi:10.3389/fpls.2021.645218)

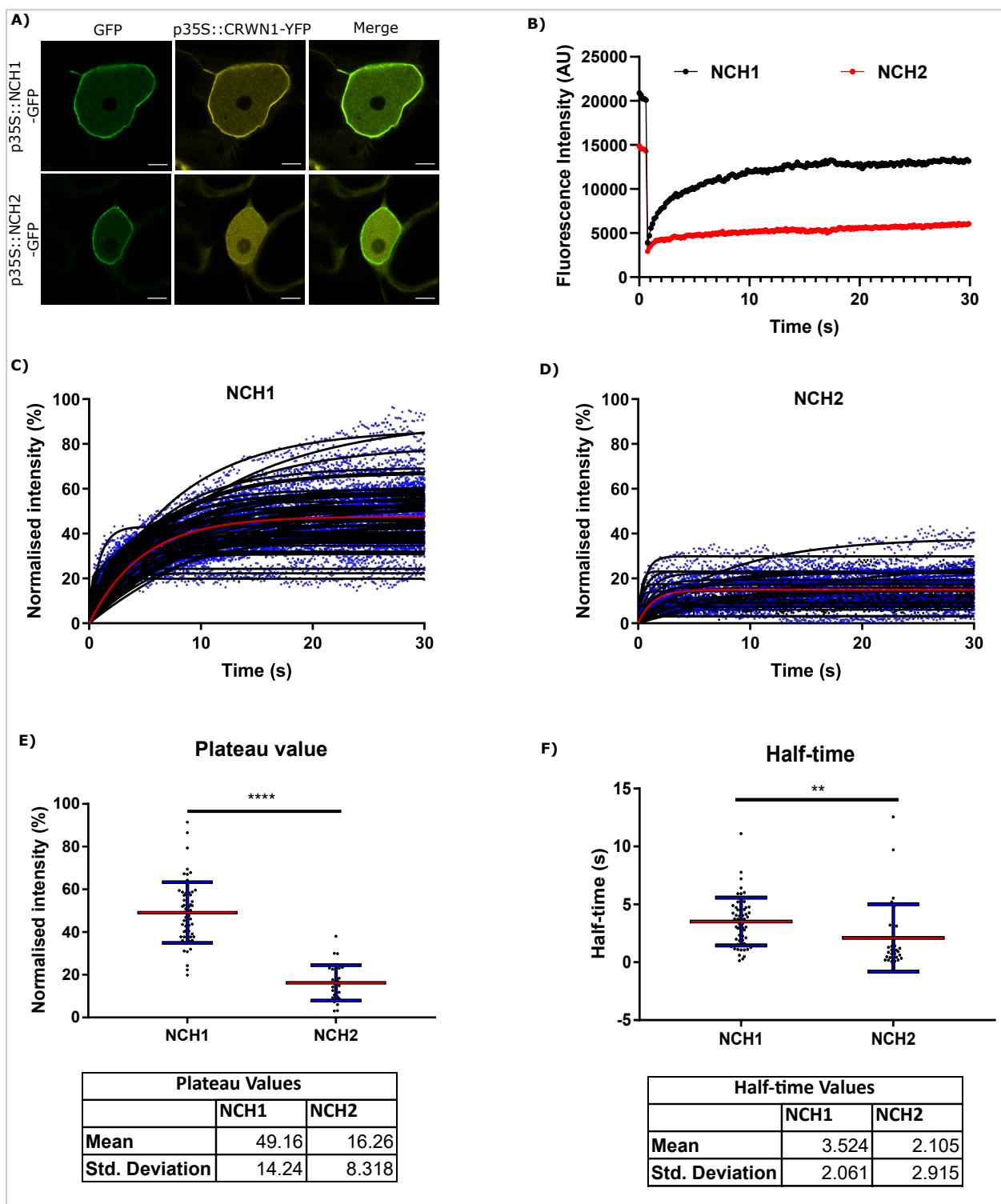

**Figure S1**  
(McKenna, Gumber, et al., submitted Dec. 2020)

Supplement: Supplementary Figure 1 — The Maize NCH1 and NCH2 proteins co-localize with their Arabidopsis homolog CRWN1. (A) Confocal live cell imaging of cells co-expressing either NCH1 or NCH2 with Arabidopsis CRWN1 shows colocalization. (B) Plot of raw FRAP intensity values over time for two representative FRAP experiments: one for NCH1 (black) and one for NCH2 (red). (C) Normalized intensity data plotted for all NCH1 FRAP curves. Blue points show individual data points, black lines show FRAP curve fits for each nucleus, and red lines show global FRAP curves produced from all datasets (As shown in Figure 1). (D) Same as panel C except for NCH2. (E) Box plots of FRAP recovery plateau values for NCH1 and NCH2. (F) Box plots of FRAP recovery half-time values for NCH1 and NCH2. For panels E and F, the individual data points from single FRAP recovery curves (black dots), the mean (red line), standard deviations (blue lines), and statistical significance (asterisks) are indicated. Students T-test performed in E and F, ∗∗p ≤ 0.01, ∗∗p ≤ 0.0001. Mean and standard deviation values are tabulated below the plots. n ≥ 30 nuclei imaged across three experimental repeats. [file Image_1.PDF]
