# Supplementary material for: Maize (Zea mays L.) Nucleoskeletal Proteins Regulate Nuclear Envelope Remodeling and Function in Stomatal Complex Development and Pollen Viability
Source: Front Plant Sci. 2021 Feb 17;12:645218. doi: 10.3389/fpls.2021.645218 (PMC7925898; doi:10.3389/fpls.2021.645218)
Supplement: Supplementary Figure 2 — Description of Sub-periphery/whole nuclei fluorescence ratio measurement. Diagram describing how the sub-periphery over whole nuclei measurement was determined using image J. Two regions of interest (ROIs) were generated, one encompassing the whole nuclei and one only sub-periphery nuclear fluorescence (yellow ROIs with hashed boundaries). The sub-periphery fluorescence value was then divided by the whole nuclei fluorescence value in order to obtain the ratio. If the majority of fluorescence is located at the periphery/nuclear envelope, this would result in a low ratio, conversely if most fluorescence was internal, this would result in a higher ratio. [file Image_2.PDF]

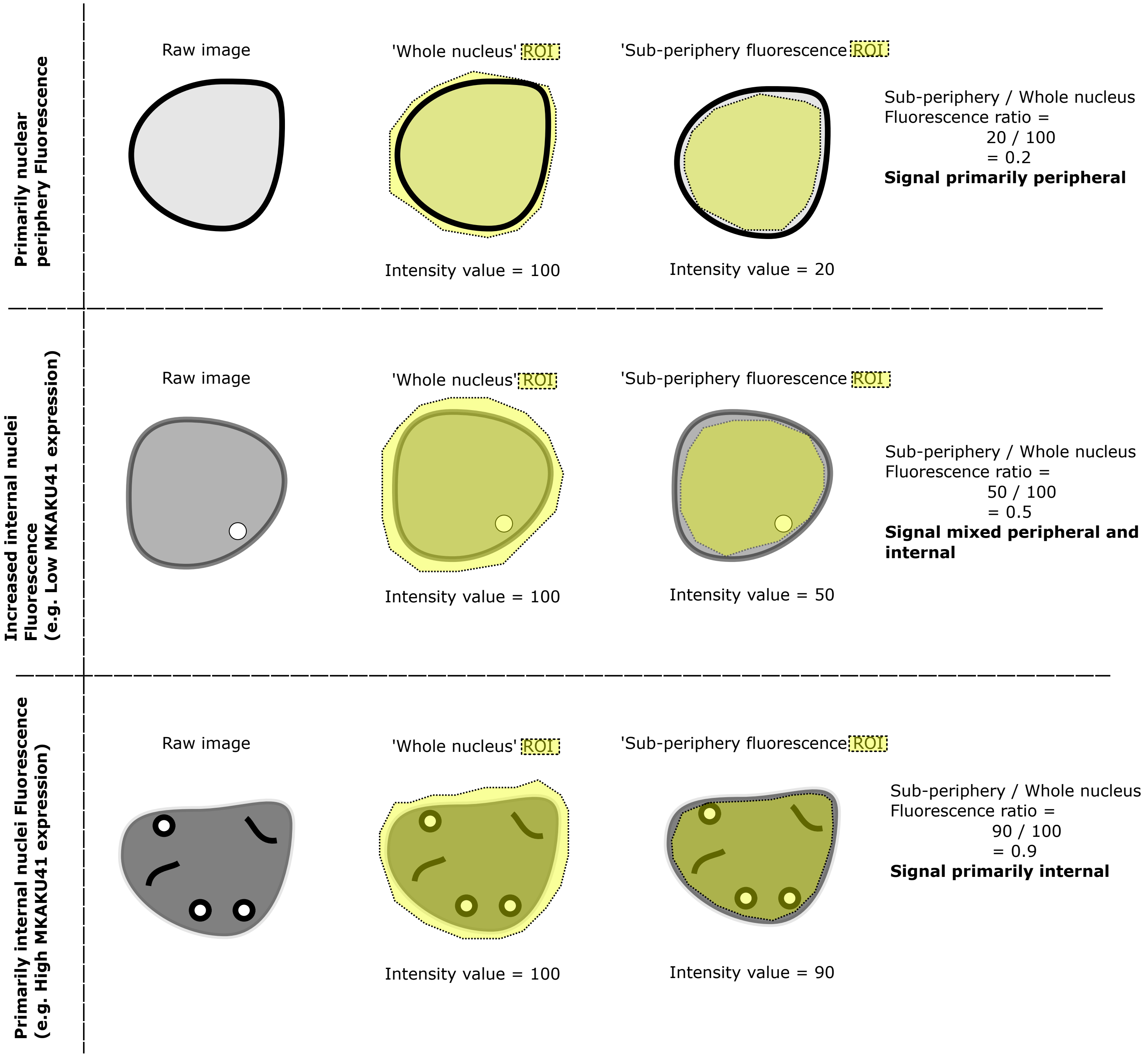
Primarily nuclear periphery Fluorescence
Raw image
'Whole nucleus' ROI
'Sub-periphery fluorescence ROI
Intensity value = 100
Intensity value = 20
Sub-periphery / Whole nucleus Fluorescence ratio = 20 / 100 = 0.2
Signal primarily peripheral
Increased internal nuclei Fluorescence (e.g. Low MKAKU41 expression)
Raw image
'Whole nucleus' ROI
'Sub-periphery fluorescence ROI
Intensity value = 100
Intensity value = 50
Sub-periphery / Whole nucleus Fluorescence ratio = 50 / 100 = 0.5
Signal mixed peripheral and internal
Primarily internal nuclei Fluorescence (e.g. High MKAKU41 expression)
Raw image
'Whole nucleus' ROI
'Sub-periphery fluorescence ROI
Intensity value = 100
Intensity value = 90
Sub-periphery / Whole nucleus Fluorescence ratio = 90 / 100 = 0.9
Signal primarily internal
