# Supplementary material for: Maize (Zea mays L.) Nucleoskeletal Proteins Regulate Nuclear Envelope Remodeling and Function in Stomatal Complex Development and Pollen Viability
Source: Front Plant Sci. 2021 Feb 17;12:645218. doi: 10.3389/fpls.2021.645218 (PMC7925898; doi:10.3389/fpls.2021.645218)
Supplement: Supplementary Figure 3 — Multiple Seq Alignment of Transcripts. (A) The 5’ UTR region and a small portion of the CDS are diagrammed as shown in Figure 5, and reversed (bottom configuration) as aligned in the multiple sequence alignment. (B) The multiple sequence alignment displays all of the RNA-seq reads with a perfect match to the 25 bp query sequence (yellow) using grep of the fastq files. All matches were converted to FASTA sequences for multiple sequence alignment. The reference genome sequence is shown at top for comparison. The sequence identifiers start with single characters for tissue (”L” for leaf; “T” for tassel), genotype (”1” for wildtype, “2” for mkaku41 homozygous mutant), or bioreplicate (”A,” “B,” or “C” for bioreplicate 1, 2, or 3, respectively), followed by unique identifier from Illumina sequence read name. The strandedness is indicated relative to the gene model, with all antisense RNAs indicated (ANTISENSE, red text). [file Image_3.PDF]

A. Gene orientation.

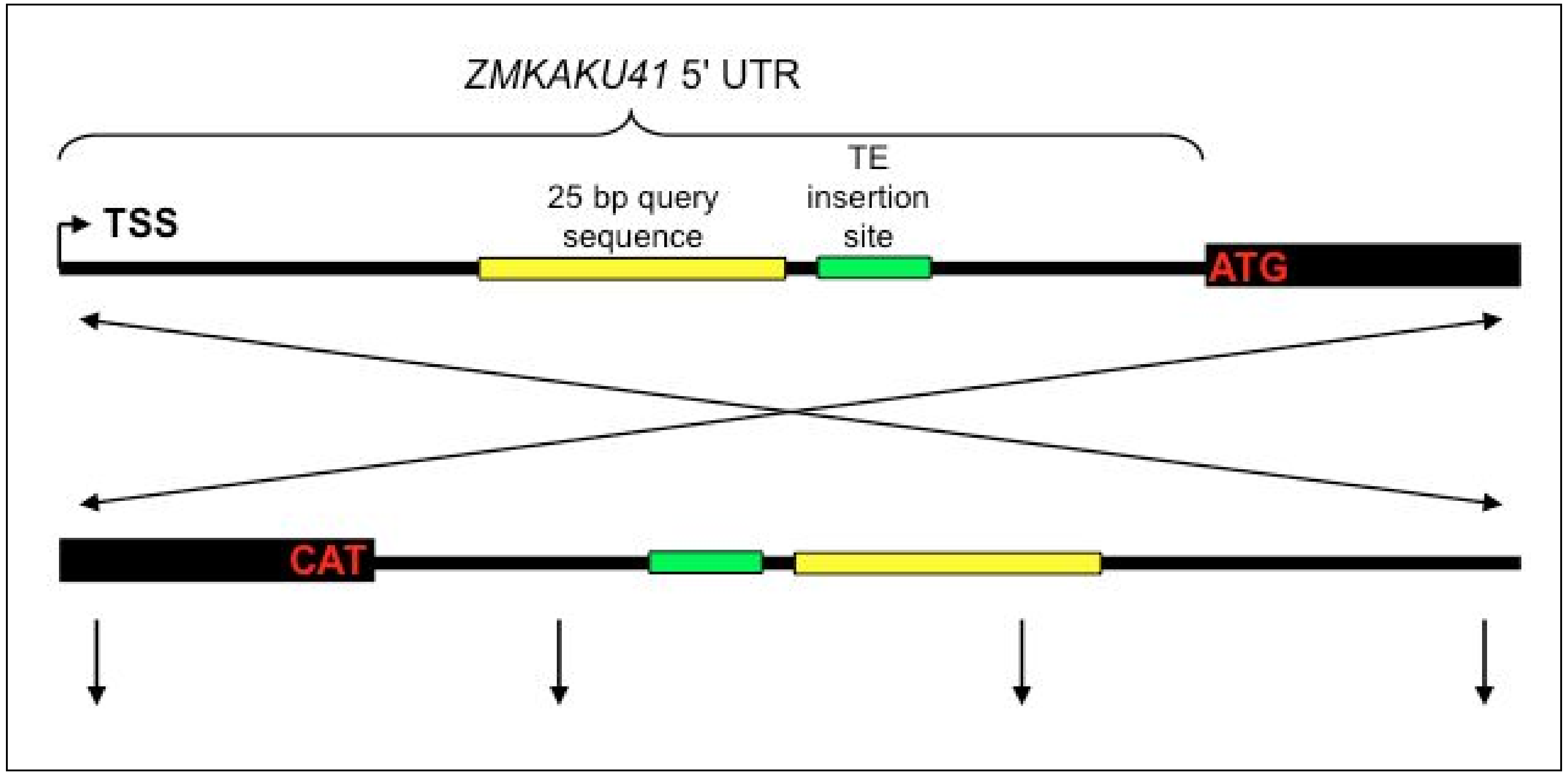

B. Multiple sequence alignment of all transcripts containing the 25 bp query sequence.

```
                                                       (<start codon)                (TE site)  (....25 bp query........)                                        (<TSS)
W22 REFERENCE SEQUENCE, T01      CGGCGATCGCCGGCGGCGAGCCCCGAAGAGGGACGCCATGGGAGGGGCGGAGGGATGGGGGGGAGGAGGAGCAAGCAGCGTGAGCGTGAGCGTGAGGCGACGAAGGATTCGAAACGGAAGCGTGAGGCGATTTTGGGAGGGGCGCCAAGAAC

>T1C_SENSE_2232_9471_24972     --CCGGCGATCGCCGGCGGCGAGCCCCGAAGAGGGACGCCATGGGAGGGGCGGAGGGATGGGGG--AGGAGGAGCAAGCAGCGTGAGCGTGAGCGTGAGGCGAC--------------------------------------------------
>T1C_SENSE_2275_13792_35618    ---CGGCGATCGCCGGCGGCGAGCCCCGAAGAGGGACGCCATGGGAGGGGCGGAGGGATGGGGG--AGGAGGAGCAAGCAGCGTGAGCGTGAGCGTGAGGCGACG-------------------------------------------------
>T1B_SENSE_2234_7500_25066     -----GCGATCGCCGGCGGCGAGCCCCGAAGAGGGACGCCATGGGAGGGGCGGAGGGATGGGGGGGAGGAGGAGCAAGCAGCGTGAGCGTGAGCGTGAGGCGACG-------------------------------------------------
>T1B_SENSE_2156_29125_22028    -------GATCGCCGGCGGCGAGCCCCGAAGAGGGACGCCATGGGAGGGGCGGAGGGATGGGGGGGAGGAGGAGCAAGCAGCGTGAGCGTGAGCGTGAGGCGACGAA-----------------------------------------------
>T1B_SENSE_2240_31114_30013    -------GATCGCCGGCGGCGAGCCCCGAAGAGGGACGCCATGGGAGGGGCGGAGGGATGGGGGGGAGGAGGAGCAAGCAGCGTGAGCGTGAGCGTGAGGCGACGAA-----------------------------------------------
>T1B_SENSE_2158_28447_2628     -------GATCGCCGGCGGCGAGCCCCGAAGAGGGACGCCATGGGAGGGGCGGAGGGATGGGGG--AGGAGGAGCAAGCAGCGTGAGCGTGAGCGTGAGGCGACGAAGG---------------------------------------------
>T1B_SENSE_2210_1344_24549     --------ATCGCCGGCGGCGAGCCCCGAAGAGGGACGCCATGGGAGGGGCGGAGGGATGGGGGGGAGGAGGAGCAAGCAGCGTGAGCGTGAGCGTGAGGCGACGAAG----------------------------------------------
>L1B_SENSE_2106_31060_8406     ----------CGCCGGCGGCGAGCCCCGAAGAGGGACGCCATGGGAGGGGCGGAGGGATGGGGG--AGGAGGAGCAAGCAGCGTGAGCGTGAGCGTGAGGCGACGAAGGATT------------------------------------------
>L1B_SENSE_2244_6985_20635     -----------GCCGGCGGCGAGCCCCGAAGAGGGACGCCATGGGAGGGGCGGAGGGATGGGGG--AGGAGGAGCAAGCAGCGTGAGCGTGAGCGTGAGGCGACGAAGGATTC-----------------------------------------
>L1C_SENSE_2133_25201_14481    --------------GGCGGCGAGCCCCGAAGAGGGACGCCATGGGAGGGGCGGAGGGATGGGGGGGAGGAGGAGCAAGCAGCGTGAGCGTGAGCGTGAGGCGACGAAGGATTCG----------------------------------------
>L1C_SENSE_2133_24207_15107    --------------GGCGGCGAGCCCCGAAGAGGGACGCCATGGGAGGGGCGGAGGGATGGGGGGGAGGAGGAGCAAGCAGCGTGAGCGTGAGCGTGAGGCGACGAAGGATTCG----------------------------------------
>T1B_SENSE_2117_24813_11553    --------------GGCGGCGAGCCCCGAAGAGGGACGCCATGGGAGGGGCGGAGGGAGGGGGGGAGGAGGAGCAAGCAGCGTGAGCGTGAGCGTGAGGCGACGAAGGATTCG----------------------------------------
>T1C_SENSE_2144_1136_27383     --------------GGCGGCGAGCCCCGAAGAGGGACGCCATGGGAGGGGCGGAGGGATGGGGG--AGGAGGAGCAAGCAGCGTGAGCGTGAGCGTGAGGCGACGAAGGATTCGAA--------------------------------------
>T1C_SENSE_2144_1118_27383     --------------GGCGGCGAGCCCCGAAGAGGGACGCCATGGGAGGGGCGGAGGGATGGGGG--AGGAGGAGCAAGCAGCGTGAGCGTGAGCGTGAGGCGACGAAGGATTCGAA--------------------------------------
>T1C_SENSE_2144_1316_27289     --------------GGCGGCGAGCCCCGAAGAGGGACGCCATGGGAGGGGCGGAGGGATGGGGG--AGGAGGAGCAAGCAGCGTGAGCGTGAGCGTGAGGCGACGAAGGATTCGAA--------------------------------------
```

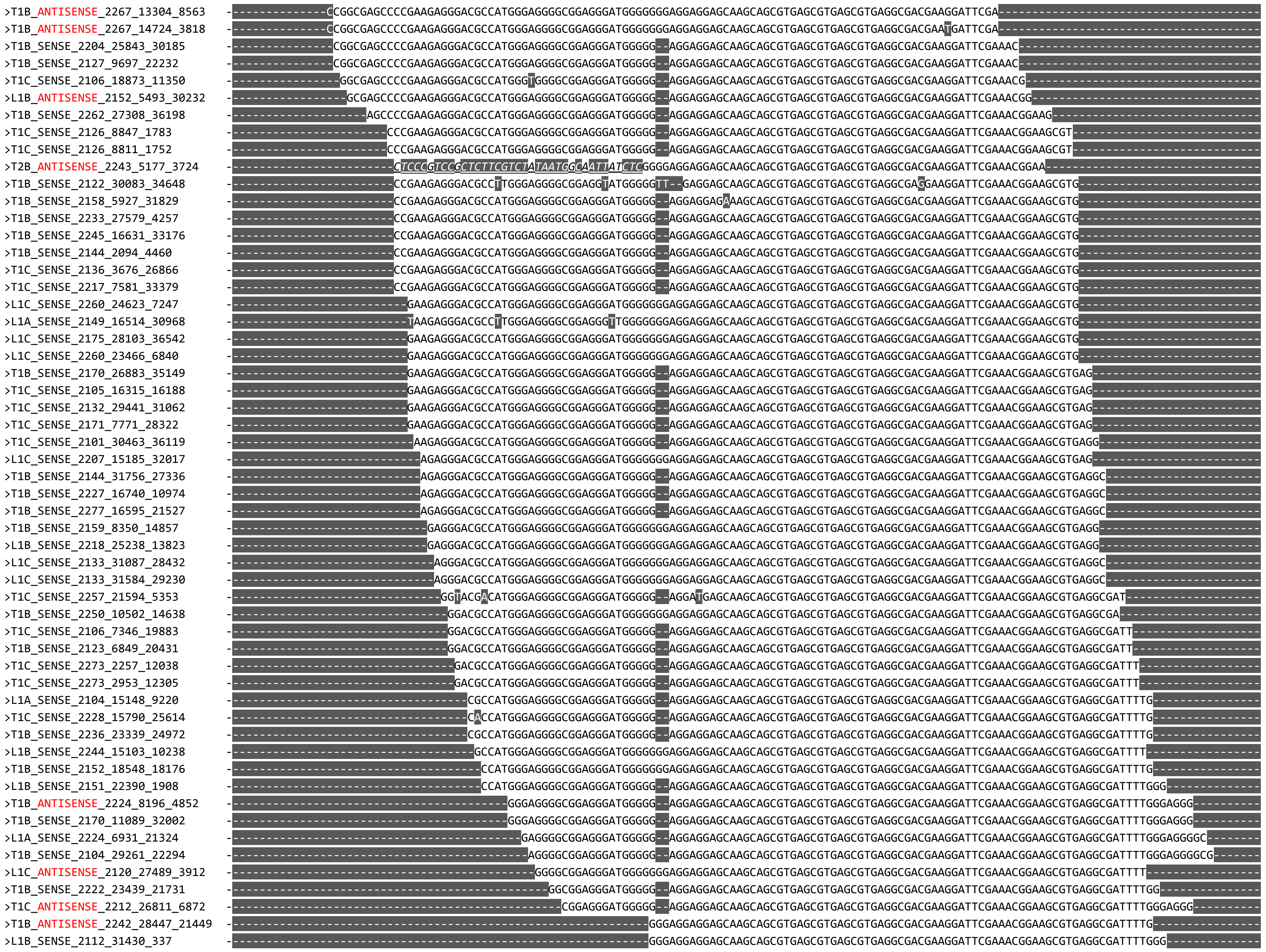

>T1B_ANTISENSE_2267_13304_8563 CCGGCGAGCCCCGAAGAGGGACGCCATGGAGGGGCGGAGGGATGGGGGGGAGGAGGAGCAAGCAGCGTGAGCGTGAGCGTGAGGCGACGAAGGATTCGA
>T1B_ANTISENSE_2267_14724_3818 CCGGCGAGCCCCGAAGAGGGACGCCATGGAGGGGCGGAGGGATGGGGGGGAGGAGGAGCAAGCAGCGTGAGCGTGAGCGTGAGGCGACGAATGATTCGA
>T1B_SENSE_2204_25843_30185 CGGCGAGCCCCGAAGAGGGACGCCATGGAGGGGCGGAGGGATGGGGG--AGGAGGAGCAAGCAGCGTGAGCGTGAGCGTGAGGCGACGAAGGATTCGAAAC
>T1B_SENSE_2127_9697_22232 CGGCGAGCCCCGAAGAGGGACGCCATGGAGGGGCGGAGGGATGGGGG--AGGAGGAGCAAGCAGCGTGAGCGTGAGCGTGAGGCGACGAAGGATTCGAAAC
>T1C_SENSE_2106_18873_11350 GGCGAGCCCCGAAGAGGGACGCCATGGTGGGGCGGAGGGATGGGGG--AGGAGGAGCAAGCAGCGTGAGCGTGAGCGTGAGGCGACGAAGGATTCGAAACG
>L1B_ANTISENSE_2152_5493_30232 GCGAGCCCCGAAGAGGGACGCCATGGAGGGGCGGAGGGATGGGGG--AGGAGGAGCAAGCAGCGTGAGCGTGAGCGTGAGGCGACGAAGGATTCGAAACGG
>T1B_SENSE_2262_27308_36198 AGCCCCGAAGAGGGACGCCATGGAGGGGCGGAGGGATGGGGG--AGGAGGAGCAAGCAGCGTGAGCGTGAGCGTGAGGCGACGAAGGATTCGAAACGGAAG
>T1C_SENSE_2126_8847_1783 CCCGAAGAGGGACGCCATGGAGGGGCGGAGGGATGGGGG--AGGAGGAGCAAGCAGCGTGAGCGTGAGCGTGAGGCGACGAAGGATTCGAAACGGAAGCGT
>T1C_SENSE_2126_8811_1752 CCCGAAGAGGGACGCCATGGAGGGGCGGAGGGATGGGGG--AGGAGGAGCAAGCAGCGTGAGCGTGAGCGTGAGGCGACGAAGGATTCGAAACGGAAGCGT
>T2B_ANTISENSE_2243_5177_3724 CTCCCGTCCGCTCTTCGTCTATAATGGCAATTATCTCGGGGAGGAGGAGCAAGCAGCGTGAGCGTGAGCGTGAGGCGACGAAGGATTCGAAACGGAA
>T1B_SENSE_2122_30083_34648 CCGAAGAGGGACGCCTTGGAGGGGCGGAGGTATGGGGGTT--GAGGAGCAAGCAGCGTGAGCGTGAGCGTGAGGCGAGGAAGGATTCGAAACGGAAGCGTG
>T1B_SENSE_2158_5927_31829 CCGAAGAGGGACGCCATGGAGGGGCGGAGGGATGGGGG--AGGAGGAGAAGCAGCGTGAGCGTGAGCGTGAGGCGACGAAGGATTCGAAACGGAAGCGTG
>T1B_SENSE_2233_27579_4257 CCGAAGAGGGACGCCATGGAGGGGCGGAGGGATGGGGG--AGGAGGAGCAAGCAGCGTGAGCGTGAGCGTGAGGCGACGAAGGATTCGAAACGGAAGCGTG
>T1B_SENSE_2245_16631_33176 CCGAAGAGGGACGCCATGGAGGGGCGGAGGGATGGGGG--AGGAGGAGCAAGCAGCGTGAGCGTGAGCGTGAGGCGACGAAGGATTCGAAACGGAAGCGTG
>T1B_SENSE_2144_2094_4460 CCGAAGAGGGACGCCATGGAGGGGCGGAGGGATGGGGG--AGGAGGAGCAAGCAGCGTGAGCGTGAGCGTGAGGCGACGAAGGATTCGAAACGGAAGCGTG
>T1C_SENSE_2136_3676_26866 CCGAAGAGGGACGCCATGGAGGGGCGGAGGGATGGGGG--AGGAGGAGCAAGCAGCGTGAGCGTGAGCGTGAGGCGACGAAGGATTCGAAACGGAAGCGTG
>T1C_SENSE_2217_7581_33379 CCGAAGAGGGACGCCATGGAGGGGCGGAGGGATGGGGG--AGGAGGAGCAAGCAGCGTGAGCGTGAGCGTGAGGCGACGAAGGATTCGAAACGGAAGCGTG
>L1C_SENSE_2260_24623_7247 GAAGAGGGACGCCATGGAGGGGCGGAGGGATGGGGGGGAGGAGGAGCAAGCAGCGTGAGCGTGAGCGTGAGGCGACGAAGGATTCGAAACGGAAGCGTG
>L1A_SENSE_2149_16514_30968 TAAGAGGGACGCCTTGGAGGGGCGGAGGGTTGGGGGGGAGGAGGAGCAAGCAGCGTGAGCGTGAGCGTGAGGCGACGAAGGATTCGAAACGGAAGCGTG
>L1C_SENSE_2175_28103_36542 GAAGAGGGACGCCATGGAGGGGCGGAGGGATGGGGGGGAGGAGGAGCAAGCAGCGTGAGCGTGAGCGTGAGGCGACGAAGGATTCGAAACGGAAGCGTG
>L1C_SENSE_2260_23466_6840 GAAGAGGGACGCCATGGAGGGGCGGAGGGATGGGGGGGAGGAGGAGCAAGCAGCGTGAGCGTGAGCGTGAGGCGACGAAGGATTCGAAACGGAAGCGTG
>T1B_SENSE_2170_26883_35149 GAAGAGGGACGCCATGGAGGGGCGGAGGGATGGGGG--AGGAGGAGCAAGCAGCGTGAGCGTGAGCGTGAGGCGACGAAGGATTCGAAACGGAAGCGTGAG
>T1C_SENSE_2105_16315_16188 GAAGAGGGACGCCATGGAGGGGCGGAGGGATGGGGG--AGGAGGAGCAAGCAGCGTGAGCGTGAGCGTGAGGCGACGAAGGATTCGAAACGGAAGCGTGAG
>T1C_SENSE_2132_29441_31062 GAAGAGGGACGCCATGGAGGGGCGGAGGGATGGGGG--AGGAGGAGCAAGCAGCGTGAGCGTGAGCGTGAGGCGACGAAGGATTCGAAACGGAAGCGTGAG
>T1C_SENSE_2171_7771_28322 GAAGAGGGACGCCATGGAGGGGCGGAGGGATGGGGG--AGGAGGAGCAAGCAGCGTGAGCGTGAGCGTGAGGCGACGAAGGATTCGAAACGGAAGCGTGAG
>T1C_SENSE_2101_30463_36119 AAGAGGGACGCCATGGAGGGGCGGAGGGATGGGGG--AGGAGGAGCAAGCAGCGTGAGCGTGAGCGTGAGGCGACGAAGGATTCGAAACGGAAGCGTGAGG
>L1C_SENSE_2207_15185_32017 AGAGGGACGCCATGGAGGGGCGGAGGGATGGGGGGGAGGAGGAGCAAGCAGCGTGAGCGTGAGCGTGAGGCGACGAAGGATTCGAAACGGAAGCGTGAG
>T1B_SENSE_2144_31756_27336 AGAGGGACGCCATGGAGGGGCGGAGGGATGGGGG--AGGAGGAGCAAGCAGCGTGAGCGTGAGCGTGAGGCGACGAAGGATTCGAAACGGAAGCGTGAGGC
>T1B_SENSE_2227_16740_10974 AGAGGGACGCCATGGAGGGGCGGAGGGATGGGGG--AGGAGGAGCAAGCAGCGTGAGCGTGAGCGTGAGGCGACGAAGGATTCGAAACGGAAGCGTGAGGC
>T1B_SENSE_2277_16595_21527 AGAGGGACGCCATGGAGGGGCGGAGGGATGGGGG--AGGAGGAGCAAGCAGCGTGAGCGTGAGCGTGAGGCGACGAAGGATTCGAAACGGAAGCGTGAGGC
>T1B_SENSE_2159_8350_14857 GAGGGACGCCATGGAGGGGCGGAGGGATGGGGGGGAGGAGGAGCAAGCAGCGTGAGCGTGAGCGTGAGGCGACGAAGGATTCGAAACGGAAGCGTGAGG
>L1B_SENSE_2218_25238_13823 GAGGGACGCCATGGAGGGGCGGAGGGATGGGGGGGAGGAGGAGCAAGCAGCGTGAGCGTGAGCGTGAGGCGACGAAGGATTCGAAACGGAAGCGTGAGG
>L1C_SENSE_2133_31087_28432 AGGGACGCCATGGAGGGGCGGAGGGATGGGGGGGAGGAGGAGCAAGCAGCGTGAGCGTGAGCGTGAGGCGACGAAGGATTCGAAACGGAAGCGTGAGGC
>L1C_SENSE_2133_31584_29230 AGGGACGCCATGGAGGGGCGGAGGGATGGGGGGGAGGAGGAGCAAGCAGCGTGAGCGTGAGCGTGAGGCGACGAAGGATTCGAAACGGAAGCGTGAGGC
>T1C_SENSE_2257_21594_5353 GGTACGACATGGAGGGGCGGAGGGATGGGGG--AGGATGAGCAAGCAGCGTGAGCGTGAGCGTGAGGCGACGAAGGATTCGAAACGGAAGCGTGAGGCGAT
>T1B_SENSE_2250_10502_14638 GGACGCCATGGAGGGGCGGAGGGATGGGGGGGAGGAGGAGCAAGCAGCGTGAGCGTGAGCGTGAGGCGACGAAGGATTCGAAACGGAAGCGTGAGGCGA
>T1C_SENSE_2106_7346_19883 GGACGCCATGGAGGGGCGGAGGGATGGGGG--AGGAGGAGCAAGCAGCGTGAGCGTGAGCGTGAGGCGACGAAGGATTCGAAACGGAAGCGTGAGGCGATT
>T1B_SENSE_2123_6849_20431 GGACGCCATGGAGGGGCGGAGGGATGGGGG--AGGAGGAGCAAGCAGCGTGAGCGTGAGCGTGAGGCGACGAAGGATTCGAAACGGAAGCGTGAGGCGATT
>T1C_SENSE_2273_2257_12038 GACGCCATGGAGGGGCGGAGGGATGGGGG--AGGAGGAGCAAGCAGCGTGAGCGTGAGCGTGAGGCGACGAAGGATTCGAAACGGAAGCGTGAGGCGATTT
>T1C_SENSE_2273_2953_12305 GACGCCATGGAGGGGCGGAGGGATGGGGG--AGGAGGAGCAAGCAGCGTGAGCGTGAGCGTGAGGCGACGAAGGATTCGAAACGGAAGCGTGAGGCGATTT
>L1A_SENSE_2104_15148_9220 CGCCATGGAGGGGCGGAGGGATGGGGG--AGGAGGAGCAAGCAGCGTGAGCGTGAGCGTGAGGCGACGAAGGATTCGAAACGGAAGCGTGAGGCGATTTTG
>T1C_SENSE_2228_15790_25614 CACCATGGAGGGGCGGAGGGATGGGGG--AGGAGGAGCAAGCAGCGTGAGCGTGAGCGTGAGGCGACGAAGGATTCGAAACGGAAGCGTGAGGCGATTTTG
>T1B_SENSE_2236_23339_24972 CGCCATGGAGGGGCGGAGGGATGGGGG--AGGAGGAGCAAGCAGCGTGAGCGTGAGCGTGAGGCGACGAAGGATTCGAAACGGAAGCGTGAGGCGATTTTG
>L1B_SENSE_2244_15103_10238 GCCATGGAGGGGCGGAGGGATGGGGGGGAGGAGGAGCAAGCAGCGTGAGCGTGAGCGTGAGGCGACGAAGGATTCGAAACGGAAGCGTGAGGCGATTTT
>T1B_SENSE_2152_18548_18176 CCATGGAGGGGCGGAGGGATGGGGGGGAGGAGGAGCAAGCAGCGTGAGCGTGAGCGTGAGGCGACGAAGGATTCGAAACGGAAGCGTGAGGCGATTTTG
>L1B_SENSE_2151_22390_1908 CCATGGAGGGGCGGAGGGATGGGGG--AGGAGGAGCAAGCAGCGTGAGCGTGAGCGTGAGGCGACGAAGGATTCGAAACGGAAGCGTGAGGCGATTTTGG
>T1B_ANTISENSE_2224_8196_4852 GGGAGGGGCGGAGGGATGGGGG--AGGAGGAGCAAGCAGCGTGAGCGTGAGCGTGAGGCGACGAAGGATTCGAAACGGAAGCGTGAGGCGATTTTGGGAGGG
>T1B_SENSE_2170_11089_32002 GGGAGGGGCGGAGGGATGGGGG--AGGAGGAGCAAGCAGCGTGAGCGTGAGCGTGAGGCGACGAAGGATTCGAAACGGAAGCGTGAGGCGATTTTGGGAGGG
>L1A_SENSE_2224_6931_21324 GAGGGGCGGAGGGATGGGGG--AGGAGGAGCAAGCAGCGTGAGCGTGAGCGTGAGGCGACGAAGGATTCGAAACGGAAGCGTGAGGCGATTTTGGGAGGGGC
>T1B_SENSE_2104_29261_22294 AGGGGCGGAGGGATGGGGG--AGGAGGAGCAAGCAGCGTGAGCGTGAGCGTGAGGCGACGAAGGATTCGAAACGGAAGCGTGAGGCGATTTTGGGAGGGGCG
>L1C_ANTISENSE_2120_27489_3912 GGGGCGGAGGGATGGGGGGGAGGAGGAGCAAGCAGCGTGAGCGTGAGCGTGAGGCGACGAAGGATTCGAAACGGAAGCGTGAGGCGATTTT
>T1B_SENSE_2222_23439_21731 GGCGGAGGGATGGGGG--AGGAGGAGCAAGCAGCGTGAGCGTGAGCGTGAGGCGACGAAGGATTCGAAACGGAAGCGTGAGGCGATTTTGG
>T1C_ANTISENSE_2212_26811_6872 CGGAGGGATGGGGG--AGGAGGAGCAAGCAGCGTGAGCGTGAGCGTGAGGCGACGAAGGATTCGAAACGGAAGCGTGAGGCGATTTTGGGAGGG
>T1B_ANTISENSE_2242_28447_21449 GGGAGGAGGAGCAAGCAGCGTGAGCGTGAGCGTGAGGCGACGAAGGATTCGAAACGGAAGCGTGAGGCGATTTTG
>L1B_SENSE_2112_31430_337 GGGAGGAGGAGCAAGCAGCGTGAGCGTGAGCGTGAGGCGACGAAGGATTCGAAACGGAAGCGTGAGGCGATTTTGGG

Legend:

A. The 5' UTR region and a small portion of the CDS are diagrammed as shown in Figure 5, and reversed (bottom configuration) as aligned in the multiple sequence alignment.

B. The multiple sequence alignment displays all of the RNA-seq reads with a perfect match to the 25 bp query sequence (yellow) using grep of the fastq files. All matches were converted to FASTA sequences for multiple sequence alignment. The reference genome sequence is shown at the top for comparison. The sequence identifiers start with single characters for tissue ("L" for leaf; "T" for tassel), genotype("1" for wildtype, "2" for mkaku41 homozygous mutant), or bioreplicate ("A", "B", or "C" for bioreplicate 1, 2, or 3, respectively), followed by unique identifier from Illumina sequence read name. The strandedness is indicated relative to the gene model, with all antisense RNAs indicated (ANTISENSE, red text).
